# Balancing selected medication costs with total number of daily injections: a preference analysis of GnRH-agonist and antagonist protocols by IVF patients

**DOI:** 10.1186/1477-7827-10-67

**Published:** 2012-08-30

**Authors:** E Scott Sills, Gary S Collins, Shala A Salem, Christopher A Jones, Alison C Peck, Rifaat D Salem

**Affiliations:** 1 Reproductive Research Division, Pacific Reproductive Center, PRC—Orange County, 10 Post, Irvine, CA, 92618, USA; 2 Centre for Statistics in Medicine, Wolfson College Annexe, University of Oxford, Oxford, UK; 3 Global Health Economics Unit and Department of Surgery, UVM College of Medicine, Burlington, VT, USA

**Keywords:** GnRH-antagonist, IVF, Preference, Patient cost, Health economics

## Abstract

**Background:**

During in vitro fertilization (IVF), fertility patients are expected to self-administer many injections as part of this treatment. While newer medications have been developed to substantially reduce the number of these injections, such agents are typically much more expensive. Considering these differences in both cost and number of injections, this study compared patient preferences between GnRH-agonist and GnRH-antagonist based protocols in IVF.

**Methods:**

Data were collected by voluntary, anonymous questionnaire at first consultation appointment. Patient opinion concerning total number of s.c. injections as a function of non-reimbursed patient cost associated with GnRH-agonist [A] and GnRH-antagonist [B] protocols in IVF was studied.

**Results:**

Completed questionnaires (*n* = 71) revealed a mean +/− SD patient age of 34 +/− 4.1 yrs. Most (83.1%) had no prior IVF experience; 2.8% reported another medical condition requiring self-administration of subcutaneous medication(s). When out-of-pocket cost for [A] and [B] were identical, preference for [B] was registered by 50.7% patients. The tendency to favor protocol [B] was weaker among patients with a health occupation. Estimated patient costs for [A] and [B] were $259.82 +/− 11.75 and $654.55 +/− 106.34, respectively (p < 0.005). Measured patient preference for [B] diminished as the cost difference increased.

**Conclusions:**

This investigation found consistently higher non-reimbursed direct medication costs for GnRH-antagonist IVF vs. GnRH-agonist IVF protocols. A conditional preference to minimize downregulation (using GnRH-antagonist) was noted among some, but not all, IVF patient sub-groups. Compared to IVF patients with a health occupation, the preference for GnRH-antagonist was weaker than for other patients. While reducing total number of injections by using GnRH-antagonist is a desirable goal, it appears this advantage is not perceived equally by all IVF patients and its utility is likely discounted heavily by patients when nonreimbursed medication costs reach a critical level.

## Background

GnRH-antagonists can serve an important role in advanced reproductive treatments, because this pharmacological approach enables the IVF sequence to be reduced from 4-5wks to <2wks, and lower overall gonadotropin consumption [1]. The attenuated stimulation associated with GnRH-antagonists also has been shown to minimize OHSS risk [2-4]. A brief stimulation regime is less physically stressful and psychologically demanding for IVF patients [5], and GnRH-antagonists have emerged as an important facilitator for this goal. But despite the praiseworthy arrival of GnRH-antagonists in the modern IVF armamentarium, it can also mean higher medication costs for those IVF patients who are required to pay for it themselves. Because medications are relatively costly and may account for more than half of the overall IVF treatment expense [6], the ‘out-of-pocket’ costs of particular agents have special relevance for fertility patients. Indeed, the laudable goal of reducing total gonadotropin amount to yield an overall lower total IVF cost (as promised by GnRH-antagonists) can only be realized when all other economic factors remain constant. And while it seems intuitive that the avoidance of unnecessary injections would appeal to IVF patients, it has never been established if this preference is conserved even when extra ‘out-of-pocket’ costs are encountered. Is the desire to reduce the number of subcutaneous injections in IVF sufficiently strong that the financial cost associated with GnRH-antagonists is irrelevant? To explore this issue, our study sought to characterize IVF patient opinion on the matter of non-reimbursed medication costs associated with either a GnRH-agonist or GnRH-antagonist protocols.

## Methods

Patients attending for reproductive endocrinology consultation at an urban IVF referral center based in southern California were invited to participate in this voluntary, anonymous questionnaire study. While this research was submitted for institutional review board (IRB) approval, it was exempted because data were gathered by voluntary questionnaire where patient identifiers were not recorded and it did not disrupt or manipulate life events. The study questionnaire was developed in a process similar to that described by Saini *et al.*[7], with input from infertility nurses, researchers, pharmaceutical executives, physicians and public members.

Patients planning a standard (non-frozen) *in vitro* fertilization (IVF) sequence with non-donor oocytes were eligible for study entry. Patients with insurance coverage for diagnostic tests & procedures, or those with any health insurance pharmacy benefit coverage for assisted fertility medications, were excluded from analysis. Any patient planning to acquire their fertility medications outside the United States were also excluded.

Study patients were informed that while multiple IVF protocols exist, no treatment approach has been consistently proven superior to any other, and specific protocols were not discussed in detail during the initial consultation. An outline of a typical IVF sequence was presented, and the need for self-administered injections was also discussed. They were advised that their subsequent ovulation induction regime and treatment timetable would be developed later, depending on findings from pending diagnostic tests. Each patient was counselled that her opinions were for research purposes only, and that the actual treatment protocol may or may not align with any preference expressed during the study. A study questionnaire was given to patients at the end of the appointment session to capture basic demographic and clinical information including patient age and duration of infertility, highest completed level of education, and history of having initiated a previous IVF cycle which included injectable gonadotropins. Relevant medical background was also queried, including history of self-administration of any other injectable medication such as insulin for diabetes, immunological agents for rheumatoid arthritis, allergy shots for allergies, etc. Because it was important to explore possible occupational familiarity with injection equipment which might bias patient opinion on self-injection, patients were asked about their work history in healthcare settings. Next, two mutually exclusive IVF treatment scenarios were contrasted. These two IVF variations ([A] = GnRH agonist beginning with oral contraceptive pill overlap in the luteal phase of the cycle immediately preceding ovarian stimulation, and [B] = GnRH antagonist commencing after ovarian stimulation) were characterized as having equivalent reproductive outcomes but different in total number of injections [8].

For the purposes of this investigation, patients were advised to make their responses with the assumption that the only difference between [A] and [B] was the total number of daily s.c. injections (estimated at 19 vs. 9 days, respectively). At our center, gonadotropins are not combined with either GnRH-agonist or antagonist, in accordance with manufacturer’s guidelines. The questionnaire concluded by asking about the relative importance of reducing number of injections as a function of additional cost that might be associated with such a reduction. The questionnaire was administered once during the initial consultation to each patient during a two month study interval. In cases where an IVF patient was accompanied by a partner, the two individuals were allowed to complete the form together if they wished. There was no time limit established to complete the questionnaire, which was filled out in a neutral, private setting at the clinic (*i.e*., not under staff observation). No penalty applied to patients who declined to participate, and those who did participate received nothing of value. Completed questionnaires were collected at patient exit and batched by front office staff for tabulation at the end of the study interval.

The calculation of medication costs for [A] and [B] was developed as follows: Preliminary “out-of-pocket” expense audits were given from randomly sampled non-donor IVF patients without insurance coverage (*n* = 49) over the six-month period immediately before study initiation. This sub-group also contributed feedback during questionnaire design, although no data collected in validation were included in the final analysis. Because receipts were not always available to document precise costs borne by patients, pharmacy records were used to verify exact the cost-to-patient associated with option [A] and [B]. Retail pharmacies (*n* = 11) serving Orange County, California (USA) were contacted to provide a “cash price” for the two prescriptions without rebates, coupons, incentives, or other special offers associated with patient purchase of either:

[A] Leuprolide acetate injection, sterile solution supplied in a 2.8 mL (multiple dose) vial, packaged as a 14-day kit (NDC 0185-7400-85).-or-Ganirelix Acetate injection, prefilled (single dose) syringe 250 μg/0.5 mL of ganirelix acetate 0.1 mg (NDC 0052-0301-51). For this investigation, it was necessary to correct unit price data for [B] by a uniform multiplier (x5) in accordance with manufacturer’s recommendation for five days’ use per each IVF cycle. Since only one 14d kit of [A] would be consumed per completed IVF cycle, a similar correction was not required. Pharmacy prices (without sales tax) for [A] and [B] were then individually tabulated and averaged to supply the “out-of-pocket” patient cost for each. Patient cost at pharmacy was calculated in 2012 U.S. dollars, without rebates, special offers, discounts or other temporary price adjustments for both protocols.

### Statistical analysis

Analysis of variance was used to assess whether there was a difference in patient age, educational level and treatment preference. Chi-square (and Fischer’s exact) test were used to evaluate the association between previous healthcare occupation and treatment preference. Cost data provided by pharmacies were compared by Student’s *t*-test. Differences where *p* < 0.05 were considered significant.

## Results

**Figure 1:**
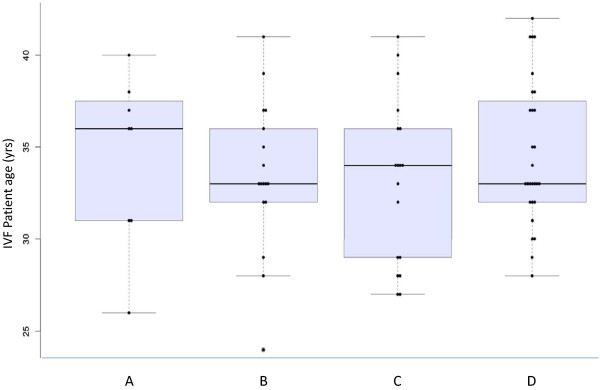
**Patient preference to reduce number of daily injections vs. IVF patient age.** Preference distribution regarding decreasing the number of daily injections and importance of reducing out-of-pocket (non-reimbursed) cost as a function of IVF patient age, where patients recorded their priority for **A** (prefer to reduce total number of daily injections), **B** (prefer to reduce out-of-pocket cost), **C** (no preference if there were no difference in cost), or **D** (prefer GnRH-antagonist, but this would be influenced by cost).

Completed questionnaires were returned by all 71 new patients eligible for entry; aggregate data provided by all patients are presented in Table 1. Distribution of IVF patient preference as a function of age is summarized in Figure 1. Mean +/− SD patient age was 34 +/− 4.1 yrs (range = 24-42 yrs), and questionnaire responses stratified by patient age are presented in Table 2. A 4-year college degree was the highest educational attainment for 33.8% of study patients; responses according to education level are summarized in Additional file 1: Table S1. Infertility duration in our sample was 2.4 +/− 1.4 (range = 0.5-8 yrs), although the mean “time trying to conceive” was not significantly different among patients who preferred the investigated treatment options (see Additional file 2: Table S2). A current healthcare occupation, or history of having ever worked in the health sector, was reported by 11 patients (15.5%), and these data are presented in Additional file 3: Table S3. In this sample, 16.9% of patients had undergone at least one prior IVF treatment at time of study entry (either at this center or elsewhere), and an analysis of questionnaire responses based on previous IVF is given in Additional file 4: Table S4. Two patients (2.8%) indicated that they currently were taking, or had ever used, any medication requiring self-injection, but responses from this subgroup were too limited to perform a separate analysis.

**Table 1:** **Aggregate responses from IVF patients (*n*** **= 71) concerning treatment preferences as a function of various cost breakpoints**

**Question**	**n (%)**
In your opinion, which factor is the **most important** regarding your upcoming fertility treatment?	
* “Reducing the total number of injections is most important to me”*	8 (11.3)
* “Reducing out-of-pocket cost is most important to me”*	17 (23.9)
* “If A and B work equally well (i.e., same pregnancy rate) then I wouldn’t care”*	18 (25.4)
* ”I would prefer B, but out-of-pocket cost would influence my choice”*	28 (39.4)
Assuming there was **no difference** in your out-of-pocket cost for A and B, what would you prefer?	
* “I would definitely prefer treatment A”*	3 (4.2)
* “If both work equally well (i.e., same pregnancy rate) then I wouldn’t care”*	29 (40.8)
* “I would definitely prefer treatment B”*	36 (50.7)
* “I don’t know”*	3 (4.2)
If reducing the total number of injections is important to you (Treatment B), and you would be willing to pay some extra for this, how much more would you be willing to pay?	
* “I would pay up to $100 more for treatment B”*	50 (70.4)
* “I would pay $100-500 more for treatment B”*	18 (25.4)
* “I would pay more than $500 for treatment B”*	2 (2.8)
* “The cost difference wouldn’t matter, because I would still want Treatment B anyway”*	1 (1.4)
Next, assume there is a difference in your ‘out-of-pocket’ cost for these two treatments. Treatment A will cost you about $260, while Treatment B will cost you about $650. What would you do based on this information?	
* “I would prefer Treatment A based on this difference”*	55 (77.5)
* “If both work equally well (i.e., same pregnancy rate) then I wouldn’t care”*	7 (9.9)
* “I would prefer Treatment B based on this difference”*	9 (12.7)
* “I don’t know”*	0

*Note*: Treatment A = GnRH-agonist, Treatment B = GnRH-antagonist.

**Table 2:** **Responses from IVF patients (*n*** **= 71) stratified by age of respondent concerning treatment preferences, as a function of various cost breakpoints**

**Question**	**Mean age (yrs)**	***p***^**1**^
In your opinion, which factor is the **most important** regarding your upcoming fertility treatment?		
* “Reducing the total number of injections is most important to me”*	33.5	0.66
* “Reducing out-of-pocket cost is most important to me”*	33.5	
* “If A and B work equally well (i.e., same pregnancy rate) then I wouldn’t care”*	33.2	
* ”I would prefer B, but out-of-pocket cost would influence my choice”*	34.6	
Assuming there was **no difference** in your out-of-pocket cost for A and B, what would you prefer?		
* “I would definitely prefer treatment A”*	35	0.016
* “If both work equally well (i.e., same pregnancy rate) then I* *wouldn’t care”*	34.2	
* “I would definitely prefer treatment B”*	34.3	
* “I don’t know”*	26.7	
If reducing the total number of injections is important to you (Treatment B), and you would be willing to pay some extra for this, how much more would you be willing to pay?		
* “I would pay up to $100 more for treatment B”*	33.5	0.025
* “I would pay $100-500 more for treatment B”*	35.7	
Next, assume there is a difference in your ‘out-of-pocket’ cost for these two treatments. Treatment A will cost you about $260, while Treatment B will cost you about $650. What would you do based on this information?		
* “I would prefer Treatment A based on this difference”*	33.5	0.176
* “If both work equally well (i.e., same pregnancy rate) then I wouldn’t care”*	35.1	
* “I would prefer Treatment B based on this difference”*	36	

*Note*: Treatment A = GnRH-agonist, Treatment B = GnRH-antagonist.

^1^Analysis of variance.

**Figure 2:**
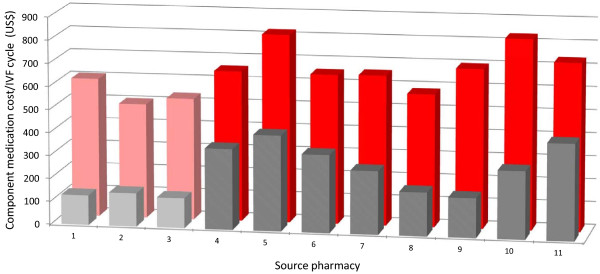
**Cost comparisons between GnRH-antagonist vs. GnRH-agonist.** Cost-to-patient (in 2012 U.S. dollars) for GnRH-antagonist (back) [**B**] and GnRH-agonist (front) [**A**] as measured in 11 retail pharmacies. Entries 1–3 were obtained from IVF specialty pharmacies, while data from sites 4–11 were derived from community pharmacies.

For all pharmacies sampled, patient cost for [A] was consistently lower that for [B], although the difference was not uniform. Static cost analysis between [A] and [B] showed that patients receiving protocol [A] would spend an average of 60.3% less for this treatment component than if they were assigned [B] during IVF. Actual mean +/− SD patient costs for [A] and [B] as determined from retail pharmacy data were $259.82 +/− 111.75 and $654.55 +/1 106.34, respectively (*p* < 0.001). Data spread for non-reimbursed pharmacy costs associated with [A] and [B] are depicted in Figure 2.

## Discussion

At least two pharmacologic approaches have been developed to prevent a premature LH surge during controlled ovarian stimulation in assisted reproduction. One technique is the long protocol of gonadotrophin-releasing hormone agonist (GnRH-a), which involves initiation of GnRH-a either in the mid-luteal or early-follicular phase of the cycle prior to ovarian stimulation. Follicular recruitment via gonadotropins is delayed until pituitary desensitization has been achieved, usually requiring 2-3wks [9]. A more recent method utilizes gonadotrophin-releasing hormone antagonists (GnRH-antagonists), which can achieve complete pituitary suppression within 4-6 h of administration [10]. Accordingly, administration of GnRH-antagonists can follow gonadotrophin administration bringing a dramatic reduction in duration of the IVF treatment cycle [11]. This substantially lowers the number of injections required during IVF, and may improve drug compliance and/or prevent errors during drug administration [12]. The use of GnRH-a and GnRH-antagonists in assisted fertility treatments has been the focus of considerable comparative research [1,13-16]. Reproductive outcomes, as well as safety and efficacy, between these two treatment approaches are thought to be similar [8,17-20] and GnRH-antagonists have joined alongside GnRH-a as mainstream therapeutic agents used widely at infertility units worldwide.

Interestingly, direct comparisons of traditional GnRH-a and newer GnRH-antagonist IVF protocols have rarely included pricing [21], and none have specifically evaluated patient opinion concerning the non-reimbursed cost for these medications. One multi-center study reported that the medicines needed for IVF account for more than half of the total treatment cost [6]. In California (and throughout the United States), GnRH-antagonists are protected by patent and command a significantly higher price compared to older GnRH-a preparations which are now available as generic substitutes. Particularly for patients whose first priority is to lower the absolute cost of IVF, if the two approaches have the same efficacy, the additional expense of a newer medication must be carefully scrutinized.

Economics is the study of scarcity, where a choice in one direction necessarily comes at the expense of what might otherwise be available in another direction. Once an IVF patient has made her choice to begin treatment, many will have already made the decision to forego services or goods that they could otherwise have afforded prior to starting IVF. Although IVF patients do, in general, prefer fewer injections, our investigation reveals that patients can sometimes make a trade-off in favor of lower out-of-pocket costs but in ways which run counter to this “fewest possible shots” axiom. This research is believed to be the first to explore this equilibrium, and while larger studies are needed, several noteworthy observations warrant discussion. First, our data align with previous studies where IVF patients responded positively to the concept of simplified IVF treatment enabled by a GnRH-antagonist [12,22-24]. Just over half of our study patients (50.7%) indicated a definite preference for GnRH-antagonist, if their ‘out-of-pocket’ cost difference between this and GnRH-a was zero. The value of this preference among IVF patients still needs refinement, but we were able to show that if the cost gap is relatively small (*e.g*., up to $100) as compared to total treatment cost, most patients (70.4%) will pay the extra expense to obtain the GnRH-antagonist associated with fewer injections. However, this study shows that when the ‘out-of-pocket’ cost difference increases, patient preference reverts to the less expensive GnRH-a product in most (77.5%) cases. Our data suggest that although most IVF patients associate greater comfort with GnRH-antagonists [25], this effect is not unlimited and will be lost when the GnRH-antagonist is perceived as too expensive.

An unexpected finding from this research was the influence of age on treatment preference if there were no differences in treatment costs, such that younger IVF patients tended not to know which treatment they would prefer compared to older IVF patients (*p* = 0.016). It could be speculated that less experienced, younger IVF patients might wish to avoid unnecessary injections and thus strongly favor GnRH-antagonist, yet this association was not supported by our study data. Because questionnaire data were stratified on the basis of IVF patients having previous or current experience working in a healthcare setting, we were able to measure differences across groups depending on this parameter. In this investigation, 11.5% of patients reported a health-related occupation where familiarity with injection techniques and equipment would be different compared to the general population. Indeed, evidence from this study suggests that IVF patients who have a health occupation background tend not to have a preference between GnRH-agonist vs. GnRH-antagonist, if there is no difference in treatment cost (*p* = 0.036). Our data also revealed that patient education level influenced treatment preference in the setting of no difference in cost, with more highly educated IVF patients tending to prefer GnRH-antagonist (*p* = 0.003). Length of time trying to conceive (infertility duration) was not associated with a preference for GnRH-antagonist in this study of IVF patients (*p* > 0.1).

Several limitations of this study should be acknowledged. Patient income was not sampled by our questionnaire, so it was impossible to stratify protocol preference by this parameter. Also, patient opinion and actual clinical decisions for IVF protocol may be quite different. Just because a patient may “dislike” a particular medication because it costs too much does not necessarily mean that it will disappear from her IVF calendar. In the context of an elective medical service like IVF, the role of patient choice is not irrelevant, however. It should be noted that the total expense of IVF represents an aggregate of numerous cost centers, and the contribution of either GnRH-a or GnRH-antagonist to overall expense might be nominal for some IVF patients. For other patients, the total quantity of gonadotropins required to complete IVF may be less when GnRH-antagonist is used [26], although precisely quantifying this effect was beyond the scope of our investigation. The established practice of pharmaceutical manufacturers giving away incentives such as free samples, rebates, coupon offers etc. for various IVF medications (including GnRH-antagonist) seems to recognize the hardships encountered by IVF patients who are struggling financially. Additionally, because our calculation of non-reimbursed medication costs was based on data from retail pharmacies serving patients in southern California, generalizing our findings to other locations should be done with caution. This investigation was not designed to follow patients through their IVF cycles to verify which protocol was actually used, or if their actual out-of-pocket cost agreed with the pre-treatment estimate. However, because our sample captured cost data from all establishments in our immediate service area plus mail-order specialty pharmacies, the ‘price to patient’ is considered accurate and the difference between actual and predicted IVF patient costs for this treatment component was probably marginal.

## Conclusions

Lack of success and psychological stress are known to be important background issues when IVF is discontinued, and not surprisingly, these factors are very strongly associated [27]. When personal economic resources are insufficient to fund further IVF treatment, this circumstance can produce its own psychological stress. Because some practice jurisdictions provide multiple cycles of IVF at zero cost to the patient, research in these settings is especially informative because it removes the economic component from the analysis of patient stress. For example, in the United Kingdom where IVF may be subsidized, population-based data revealed a relatively high attrition rate whereby IVF patients on average tend to either succeed with a pregnancy or drop-out from treatment altogether after only two completed cycles (Jones CA, 2006; DPhil dissertation, University of Oxford). Another IVF study in a Scandinavian healthcare setting reported that among 450 couples not achieving live birth, 208 completed their subsidized cycles, but 242 discontinued IVF even though the patient would have paid almost nothing (*i.e*., payment would have been made by the state). Reasons for stopping treatment included psychological stress (26%), poor prognosis (25%), spontaneous pregnancy (19%), physical burden (6%), serious disease (2%), and other reasons (7%) [28]. Of note, some locations within USA do mandate insurance coverage for IVF and when these patients were sampled similar attrition trends have been observed [29]. We anticipate future investigation into the behavioral economics of IVF patient preferences and value-based incentives in elective fertility treatments can clarify our initial findings.

## Competing interests

The authors declare that they have no competing interests.

## Authors’ contributions

ESS was principal investigator and conceptualized the study; GSC was chief statistician and assisted with study design; SAS and ACP were physicians contributing to data collection and manuscript preparation; CAJ was health economist who assisted in manuscript development; RDS was co-investigator and division director. All authors read and approved the final manuscript.

## Supplementary Material

Additional file 1 Table S1 Responses from IVF patients (*n* = 71) stratified by highest completed level of education. Click here for file

Additional file 2 Table S2 Responses from IVF patients (*n* = 71) stratified by length of time trying to conceive. Click here for file

Additional file 3 Table S3 Responses from IVF patients (*n* = 71) with a past or present health occupation. Click here for file

Additional file 4 S4 Responses from IVF patients (*n* = 71) stratified by previous IVF experience. Click here for file
