# Supplementary material for: Balancing selected medication costs with total number of daily injections: a preference analysis of GnRH-agonist and antagonist protocols by IVF patients
Source: Reprod Biol Endocrinol. 2012 Aug 30;10:67. doi: 10.1186/1477-7827-10-67 (PMC3447708; doi:10.1186/1477-7827-10-67)
Supplement: Additional file 2 — Table S2 Responses from IVF patients (n = 71) stratified by length of time trying to conceive. [file 1477-7827-10-67-S2.doc]

**Supplemental Table S2. Responses from IVF patients (*n***=71) stratified by length of time trying to conceive, regarding treatment preferences as a function of various cost breakpoints.

| Question | Infertility duration (yrs) | *p*^1^ |
| --- | --- | --- |
|  |  |  |
| In your opinion, which factor is the **most important** regarding your upcoming fertility treatment? |  |  |
| *“Reducing the total number of injections is most*  *important to me”* | 2.2 | 0.88 |
| *“Reducing out-of-pocket cost is most important to me”* | 2.4 |
| *“If A and B work equally well (i.e., same pregnancy*  *rate) then I wouldn’t care”* | 2.5 |
| *”I would prefer B, but out-of-pocket cost would*  *influence my choice”* | 2.2 |
|  |  |  |
| Assuming there was **no difference** in your out-of-pocket cost for A and B, what would you prefer? |  |  |
| *“I would definitely prefer treatment A”* | 0.8 | 0.14 |
| *“If both work equally well (i.e., same pregnancy rate)*  *then I wouldn’t care”* | 2.6 |
| *“I would definitely prefer treatment B”* | 2.3 |
| *“I don’t know”* | 1.7 |
|  |  |  |
| If reducing the total number of injections is important to you (Treatment B), and you would be willing to pay some extra for this, how much more would you be willing to pay? |  |  |
| *“I would pay up to $100 more for treatment B”* | 2.4 | 0.78 |
| *“I would pay $100-500 more for treatment B”* | 2.3 |
|  |  |  |
| Next, assume there is a difference in your ‘out-of-pocket’ cost for these two treatments. Treatment A will cost you about $260, while Treatment B will cost you about $650.  What would you do based on this information? |  |  |
| *“I would prefer Treatment A based on this difference”* | 2.4 | 0.29 |
| *“If both work equally well (i.e., same pregnancy rate)*  *then I wouldn’t care”* | 2.9 |
| *“I would prefer Treatment B based on this difference”* | 1.8 |
|  |  |  |

*Note*: Treatment A = GnRH-agonist, Treatment B = GnRH-antagonist

^1^ Analysis of variance
